# Patients’ palliative care needs in rural health and a proposal for palliation services

**DOI:** 10.4102/phcfm.v17i1.4866

**Published:** 2025-04-28

**Authors:** Deidre Pretorius, Lesley G. Mahole

**Affiliations:** 1 Department of Family Medicine and Primary Care, Faculty of Health Sciences, University of the Witwatersrand, Johannesburg, South Africa

**Keywords:** palliative care, rural, model for services, family physician, primary care, end of life care, social work, multidisciplinary team

## Abstract

Few patients and their families receive palliative care (PC), and if provided, it is usually in the end stages of the disease. In the past, these services were rendered by non-governmental organisations (NGOs), but after the dedicated South African palliative care policy was released, the responsibility of PC service delivery is at the provincial level. Department of Health’s National Policy Framework and Strategy on PC was a major step forward in palliation; however, the services are not yet reaching the rural areas in North West province. This article highlights the need for PC for patients and their families in rural health. A model is suggested to facilitate these services under the leadership of a family physician.

## Background

South Africa’s healthcare system is overburdened with both infectious and non-communicable diseases in both rural and urban areas.^1^ Most of these life-limiting or life-threatening conditions that form part of the quadruple burden of disease, require palliative care (PC) services.^2,3^ It is estimated that globally, only 14% of people who require PC services will receive PC.^4^ Services are rendered for various conditions, for example, acquired immunodeficiency syndrome (AIDS), cancer, cardiovascular disease, chronic liver disease, congenital anomalies, diabetes, disability adjusting life expectancy, multiple sclerosis, neurological disease and rheumatoid arthritis.^2,3,5^

**FIGURE 1:**
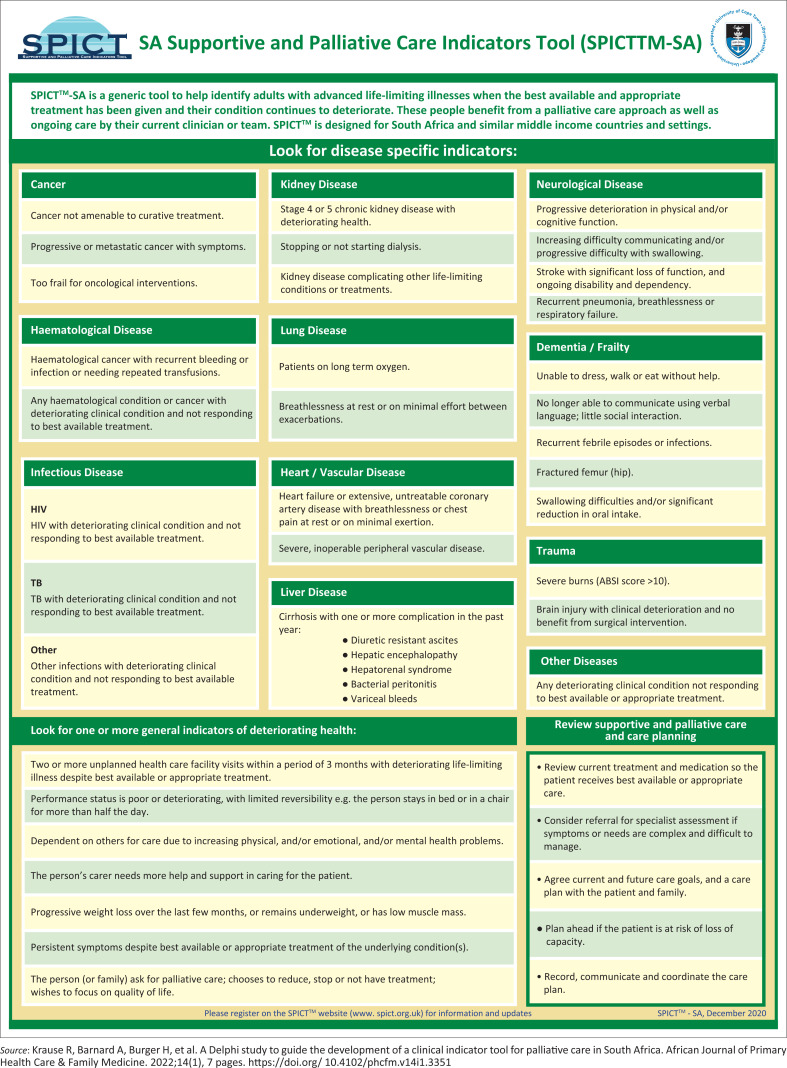
SA Supportive and Palliative Care Indicators Tool (SPICTTM-SA).

An expert advisory group in South Africa developed a tool SPICT SA to provide equitable access to PC (See Figure 1).^6^

Palliative care aims to improve the quality of life of persons and their families who are facing problems associated with life-limiting or life threatening illnesses.^2,3,5^ It prevents and relieves suffering through the early identification, impeccable assessment and treatment of pain and other problems, whether physical, psychosocial, or spiritual.^4^ These services can be delivered in several ways, namely home-based care, mobile outreach services, outpatient care, inpatient care facilities, hospital-based care teams, day care services, frail care and other care homes, workplace programmes, and correctional services.^2^

## Need assessment in a rural setting in North West province

Developed countries also experience that rural populations have higher mortality, low socio-economic status and seldom access to PC services.^6^ Clinical training in PC and dedicated staff, as well as a lack of resources are common challenges preventing PC service delivery in rural areas.^6^ The current PC structure in North West relies on down referrals from the tertiary hospital for services available in the primary care setting. Patients are often not informed about the end of life diagnoses and also do not have knowledge to ask for PC services.^7,8^ The North West province and health districts have autonomy on spending of budgets and initiate improvements favouring urban areas, which contributed to interprovincial variations in health outcomes.^9^ Ventersdorp does not have an official dedicated PC programme and thus using an adopted standard operating procedure (SOP) from urban areas not aligned for the specific needs of the patients in the rural areas.

Despite the Department of Health’s National Policy Framework and Strategy on PC 2017–2022 document,^5^ services are still inadequate in rural areas. Palliative care services are as Association of Palliative Care Centres (APCC) is a not-for-profit company^11^ thus, personnel numbers and finances may not allow them to travel far to render regular services in rural areas. Furthermore, they cannot render a service if a medical practitioner does not refer the patient. Medical practitioners have an advocacy role to ensure PC services in the public health sector.^2^

In 2019, Mahole G.L. did a survey (Perceptions of terminally ill patients concerning the palliative care they receive in rural primary healthcare) in Ventersdorp and surrounding rural areas, using a researcher administered Problems and Needs in Palliative Care questionnaire – short version (PNPC-sv).^11^ The study focussed on patients with cancer being referred from a tertiary hospital in North West Matlosana Health District to a rural setting in Ventersdorp for end-of-life care.^12^ Although not a big sample (*n* = 40), it clearly depicted the unmet needs of patients.^12^ The participants consisted of 16 men and 24 women between the ages of 39 and 79. Thirty patients (75%) were dependent on children and spouses for caregiving. All the patients had psychological challenges such as a depressed mood, worried about physical suffering and feared progression of disease, the unpredictability of the future, and difficulties to share emotions.^12^ The need for information trumped all other needs, with 97% of the patients asking for information. Of the 81% of the patients who experienced these problems with PC, 92% wanted professional help rather than assistance and care from relatives.^12^ Thirty-two patients (80%) were of the opinion that the services they received from the subdistrict were not satisfactory, while the others did not expect better services. When comparing the needs of men and women, women in this study were more likely to express a need for physical care (*p* ≤ 0.027), and social assistance (*p* ≤ 0.019). Patients in professional care (old age home) did not express a need for professional help regarding autonomy or spiritual issues. The conclusion was that health services are failing patients in the rural areas of Ventersdorp, North West province. These patients were sent home to an impoverished area without resources or healthcare workers with optimal PC knowledge and experience.

Learning from other successful models was the first priority. Community-based programmes proved to be successful in rural areas across three states in the United States.^6^ Western Cape South Africa, has an integrated model where the regional hospital’s multidisciplinary team, do home visits and short course training rendering effective services for PC in a rural area.^14^ One has to take into account that both these models were implemented in areas not as low-resourced as the North West province where people struggle to survive poverty every day. Other services can be incorporated, for example, the APCC – a national NGO for PC. They render services with volunteers of which 90% is home-based care.^10^ Nationally, APCC has four service outlets in North West province aiming to give care to a broader population of 3 852 976 individuals in North West.^10,14^ This service is not available to people in Ventersdorp and surrounding rural areas.

**FIGURE 2:**
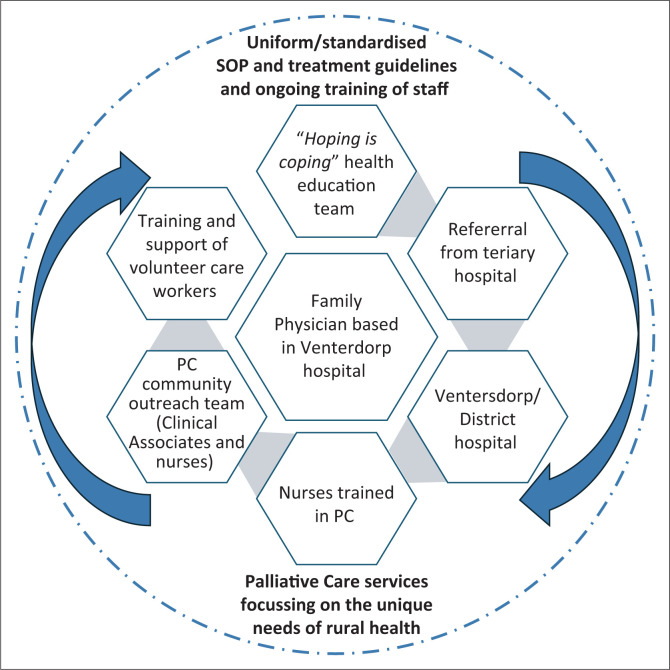
Proposed service model for palliative care in a rural setting in North West province.

Based on the research outcome, a meeting was set with Ventersdorp subdistrict manager, District Director and Chief Director to address the issue of insufficient services to patients needing palliative care. The need for PC services is recognised, and there is a vision to provide the service. A suggestion was made to establish a palliative care unit at Ventersdorp District hospital. A combination of the community-based PC and integrated model was adopted as a palliative care service model (Figure 2). It will start with a SOP based on needs of the inhabitants of the rural area in and surrounding Ventersdorp (Figure 2). The plan is to bring political leadership on board as the planned PC unit would change service package at primary healthcare level and would also have financial implications. This would also require consultations with clinic and hospital boards as well as community representations to help with creating awareness regarding the need and development of the unit.

## Proposed palliative care service model

The proposed model (Figure 2) has the family physician at the centre in a district hospital leading the PC programme and development of a specific SOP, both clinical management and referral, which will link the tertiary hospital services in the referral pathway of the patient to primary care services. A multidisciplinary team will be involved with the ‘Hoping is Coping’ programme to do health education and render support. This programme will be led by a clinical social worker and clinician where newly diagnosed patients and their families are orientated on the diagnosis, treatment modalities and where they can find help and support for challenges they may experience. The idea is that this already starts early in the diagnosis before the patient develops complications or reaches the end of life phase. When a patient is diagnosed late, the patient and family can still join such a group. Nurses trained in palliative care will monitor the patients in hospital but also supervise the community outreach teams. These outreach teams will not only deliver person-centred care but also train volunteers in home-based care.

### Ethical Considerations

Ethical clearance to conduct this study was obtained from the University of the Witwatersrand Human Research Ethics Committee (No. M170107) and the Department of Health Ventersdorp Sub District dated 19 May 2017.

## Conclusion

The advantages of this proposed service model focused on the needs of patients in rural settings are multidisciplinary support for the patient, community awareness and education, capacitating of carers and support for the healthcare team to decrease the risk for vicarious trauma. Department of Health prioritised PC. When staff is available, appointments can facilitate the implementation of PC services in rural areas.

The author, D.P., serves as an editorial board member of this journal. D.P. has no other competing interests to declare.

This article is partially based on the author L.G.M.s thesis titled “Perceptions of terminally ill patients concerning the palliative care they receive in rural primary health care” towards the degree of Master of Medicine in Family Medicine in the Faculty of Health Sciences, University of the Witwatersrand, South Africa in 2020, with supervisor Dr C. Lion- Cachet and Co-Supervisor: Mrs D. Pretorius. It is available here: https://wiredspace.wits.ac.za/items/83774d06-2060-4213-ae99-3980d75b2d1a/full.
